# Physiologically Relevant Concentrations of Dolutegravir, Emtricitabine, and Efavirenz Induce Distinct Metabolic Alterations in HeLa Epithelial and BV2 Microglial Cells

**DOI:** 10.3389/fimmu.2021.639378

**Published:** 2021-05-20

**Authors:** Joseph W. George, Jane E. Mattingly, Nashanthea J. Roland, Cassandra M. Small, Benjamin G. Lamberty, Howard S. Fox, Kelly L. Stauch

**Affiliations:** Department of Neurological Sciences, University of Nebraska Medical Center, Omaha, NE, United States

**Keywords:** antiretrovirals, dolutegravir, emtricitabine, glycolysis, microglia, mitochondria

## Abstract

Microglia, the resident brain phagocytes, likely play a key role in human immunodeficiency virus (HIV) infection of the central nervous system (CNS) and subsequent neuropathogenesis; however, the nature of the infection-induced changes that yield damaging CNS effects and the stimuli that provoke microglial activation remains elusive, especially in the current era of using antiretroviral (ARV) drugs for ARV therapy (ART). Altered microglial metabolism can modulate cellular functionality and pathogenicity in neurological disease. While HIV infection itself alters brain energy metabolism, the effect of ARV drugs, particularly those currently used in treatment, on metabolism is understudied. Dolutegravir (DTG) and emtricitabine (FTC) combination, together with tenofovir (TAF or TDF), is one of the recommended first line treatments for HIV. Despite the relatively good tolerability and safety profile of FTC, a nucleoside reverse transcriptase inhibitor, and DTG, an integrase inhibitor, adverse side effects have been reported and highlight a need to understand off-target effects of these medications. We hypothesized that similar to previous ART regimen drugs, DTG and FTC side effects involve mitochondrial dysfunction. To increase detection of ARV-induced mitochondrial effects, highly glycolytic HeLa epithelial cells were forced to rely on oxidative phosphorylation by substituting galactose for glucose in the growth media. We assessed ATP levels, resazurin oxidation-reduction (REDOX), and mitochondrial membrane potential following 24-hour exposure (to approximate effects of one dose equivalent) to DTG, FTC, and efavirenz (EFV, a known mitotoxic ARV drug). Further, since microglia support productive HIV infection, act as latent HIV cellular reservoirs, and when dysfunctional likely contribute to HIV-associated neurocognitive disorders, the experiments were repeated using BV2 microglial cells. In HeLa cells, FTC decreased mitochondrial REDOX activity, while DTG, similar to EFV, impaired both mitochondrial ATP generation and REDOX activity. In contrast to HeLa cells, DTG increased cellular ATP generation and mitochondrial REDOX activity in BV2 cells. Bioenergetic analysis revealed that DTG, FTC, and EFV elevated BV2 cell mitochondrial respiration. DTG and FTC exposure induced distinct mitochondrial functional changes in HeLa and BV2 cells. These findings suggest cell type-specific metabolic changes may contribute to the toxic side effects of these ARV drugs.

## Introduction

While antiretroviral therapy (ART) has led to tremendous reductions in morbidity and mortality associated with human immunodeficiency virus (HIV), antiretroviral (ARV) drugs are associated with a variety of peripheral and central adverse events (1, 2). Furthermore, as life expectancy for individuals living with HIV has increased, the long-term safety of ARV drugs has garnered increasing attention. Long-term complications continue to occur in HIV-infected individuals, despite the widespread use of ART, and can be related to the virus itself or to adverse effects of ARV drugs (3–5). The precise mechanisms of ARV toxicity are not fully understood, but in the case of efavirenz (EFV), we (6, 7), and others (8–12) have found effects on mitochondria.

Mitochondria, which produce energy for the cell *via* oxidative phosphorylation, have long been known to be affected by certain ARV drugs (13–16). In particular, the nucleoside reverse transcriptase inhibitors (NRTIs) affect mitochondrial function, and it has been proposed that NRTI mitochondrial toxicity may underlie the wide spectrum of clinical side effects caused by these agents (13). Similarly, neuro- and hepatotoxic effects of EFV are likely due to mitochondrial toxicity (6, 8, 11, 12, 17, 18). While EFV use is decreasing, it is important to understand if physiologically relevant concentrations of currently used ARV drugs affect mitochondrial functions. As dolutegravir (DTG) and emtricitabine (FTC, together with tenofovir (TAF or TDF)) combination is one of the first line treatments for HIV, these drugs were studied. While ARVs can affect many organs, the entry into the central nervous system (CNS) is frequently limited. DTG and FTC are both CNS penetrant, as is EFV, an ARV drug with known mitotoxic effects (19, 20).

Chronic neuroinflammation driven by glial activation is commonly implicated as a contributing factor to neurodegeneration and cognitive impairment in HIV-infected individuals (21, 22). Microglia, the CNS-resident macrophages, support productive HIV infection and likely play a major role in subsequent neurotoxicity (23, 24). Indeed, microglial activation during HIV infection is suggested to contribute to HIV-associated neurocognitive disorders (HAND) development. Modulation of microglial metabolism is increasingly recognized as a mechanism underlying activation of microglia in neurodegenerative diseases (24–26). HIV infection itself disturbs brain bioenergetics and metabolic disturbances in the CNS exist despite ART (27).

While all medications have potential side effects, it is important that these do not initiate or worsen any of the problems that HIV infection causes in the brain or elsewhere in the body. We hypothesized that similar to previous ART regimen drugs, DTG and FTC side effects involve mitochondrial dysfunction, and have the potential themselves to alter immunometabolism. The present study investigates changes in several metabolic parameters (ATP levels, resazurin oxidation-reduction (REDOX), mitochondrial membrane potential, and bioenergetics) in a non-CNS- and a CNS-derived cell line after treatment with DTG, FTC, or EFV. To increase detection of and ascribe the effects to ARV-induced mitochondrial toxicity, we employed the glucose-galactose assay. This assay, often utilized to preclinically screen for drug-induced mitochondrial dysfunction (28–30), involves culturing heavily glycolytic cells, such as HepG2 hepatoma and HeLa epithelial cells, in media where glucose has been replaced with galactose to force the cells to rely on mitochondrial oxidative phosphorylation for the production of ATP, increasing their sensitivity to mitotoxicants. Thus, HeLa cells were chosen for an initial screen for DTG- and FTC-induced mitochondrial changes, then upon identification of metabolic changes upon exposure to these ARV drugs additional experiments were performed in the well-used and characterized BV2 microglial cell line to uncover the relevance of these changes to immunometabolism. The ARV drugs were assessed separately to uncover the metabolic impact of each individual ARV drug, which is important as clinicians strive to use less toxic cART regimens.

## Materials and Methods

### Cell Culture

HeLa and BV2 cells were cultured in DMEM (Gibco, Gaithersburg, MD) containing 10% FBS (Corning, Corning, New York), 1% PEN/STR (Gibco), and 4 mM L-glutamine (Gibco) in a humidified incubator with 5% CO_2_ at 37°C. For Seahorse, cells were seeded at 15,000 cells/well in poly-D-lysine (PDL, Sigma, St. Louis, MO) coated 96-well microplates (Agilent Technologies, Santa Clara, CA). For ATP, AlamarBlue, and LDH, cells were seeded at 10,000 cells/well in PDL coated 96-well tissue culture plates. For LIVE/DEAD flow cytometry, cells were seeded at 150,000 cells/well in 12-well tissue culture plates. For JC-1 and TMRE flow cytometry, cells were seeded at 350,000 cells/well in 6-well tissue culture plates. HeLa cells were obtained from ATCC (Manassas, VA). BV2 cells were a kind gift from Dr. Shilpa Buch (University of Nebraska Medical Center), originally provided by Dr. Sanjay Maggirwar (George Washington University).

### Antiretroviral Treatments

DTG was from BOC Sciences (Shirley, NY). FTC and EFV were from NIH AIDS Reagent Program, NIAID (Germantown, MD). Stock solutions were made in DMSO (Sigma) and stored at -20°C. ARV drugs were prepared in DMEM containing 3% FBS and 4 mM L-glutamine, with glucose and without glucose, supplemented with 10 mM galactose (Sigma). 24-hours after plating, cells were treated with DTG (43 and 4300 nM), FTC (441 and 44100 nM), or EFV (44 and 4400 nM) at concentrations consistent with reported CSF levels (20, 31, 32) and a 100x higher concentration (all wells contained 0.1% DMSO). 24-hours after ARV treatments, the cells were used for experiments. For the ATP, REDOX, and Cytotoxicity experiments: media alone, vehicle alone, and 2% Triton X-100 (Fisher Scientific, Hampton, NH) containing wells were included in each experiment; all treatments were done in six (media, Triton X-100, and ARV drug treatments) to twelve (vehicle control) technical replicate wells, represent readings from six to twelve wells of the same plate that were averaged to one value, with four to five biological replicates (cells plated on different plates that were derived from different batches).

### ATP Measurements

ATP levels were measured using the CellTiter-Glo Luminescent Cell Viability Assay (Promega, Madison, WI). Luminescence was measured using a Synergy HTX Multi-Mode Microplate Reader (BioTek, Winooski, VT). Percentage of vehicle control was calculated as follows: (experimental treatment – 2% Triton X-100)/(vehicle – 2% Triton X-100) x 100.

### REDOX Measurements

Resazurin was used as an oxidation-reduction (REDOX) indicator (AlamarBlue Cell Viability Reagent, Invitrogen, Waltham, MA). Fluorescence at 560/15 nm and 590/20 nm was measured using a Synergy HTX Multi-Mode Microplate Reader. Percentage of vehicle control was calculated as follows: (experimental treatment – 2% Triton X-100)/(vehicle – 2% Triton X-100) x 100.

### Cytotoxicity Detection

Cytotoxicity was measured using the Lactate Dehydrogenase (LDH) Cytotoxicity Detection Kit (Roche, Indianapolis, IN). Absorbance at 490 nM was measured using a Synergy HTX Multi-Mode Microplate Reader. LDH percent cytotoxicity was calculated as follows: (experimental treatment – media alone)/(2% Triton X-100 – media alone) x 100.

### Cell Viability

Cell viability was determined using the LIVE/DEAD™ Fixable Blue Dead Cell Stain Kit (Invitrogen). As a positive control, cells were heat shocked (agitated at 65°C for 5 minutes). The cells were analyzed on a BD LSR Fortessa X-50 Cell Analyzer (BD Biosciences, San Jose, CA) with UV excitation using the 427/25 emission filters.

### Mitochondrial Membrane Potential

Mitochondrial membrane potential was measured using the MitoProbe JC-1 Assay Kit for Flow Cytometry (Invitrogen). As a positive control, cells were treated with 20 µM CCCP (Sigma) for 30 minutes. The cells were analyzed on a BD LSR Fortessa X-50 Cell Analyzer with 405 nm excitation using 586/15BP and 525/50BP emission filters (33). Additionally, the TMRE-Mitochondrial Membrane Potential Assay Kit (Abcam) was used. As a positive control, HeLa cells were treated with 20 µM FCCP (Abcam), while BV2 cells were treated with 10, 20, and 40 µM FCCP for 30 minutes. Cells were then treated with 400 nM TMRE for 30 minutes and analyzed using a BD LSR Fortessa X-50 Cell Analyzer with 488 nm excitation using 586/15BP emission filters, per manufacturer’s instruction.

### Bioenergetics

Oxygen consumption rate (OCR) and extracellular acidification rate (ECAR) were measured at 37°C using the XFe96 Extracellular Flux analyzer (Agilent Technologies). 24-hours after ARV treatments, the cells were washed once with XF assay medium containing 4 mM L-glutamine and 25 mM glucose. Then XF assay medium containing ARV drugs was replaced and the cells were placed in a non-CO_2_ incubator at 37°C for 1-hour prior to the assay. Three baseline measurements of OCR and ECAR were recorded prior to sequential injection of oligomycin (O, 1 μM, ATP synthase complex inhibitor), carbonyl-cyanide-4-phenylhydrazone (FCCP, F, 300 nM, ATP synthesis uncoupler), rotenone (R, 2 μM, complex I inhibitor) and antimycin A (AA, 2 μM, complex III inhibitor). After completion of the assay, total protein was isolated from individual wells and quantified using a BCA Protein Assay Kit (Pierce Biotechnology, Waltham, MA). Each well was normalized to μg of protein. All treatments were done in six technical replicate wells, represent readings from six wells of the same microplate that were averaged to one value, with five biological replicates (cells plated on different microplates that were derived from different batches).

### Statistical Analysis

ATP, Alamar, and LDH data were acquired in Gen5 software (BioTek) and processed in Microsoft Excel (Microsoft Corporation, Redmond, WA). Outlier exclusion was applied to data points (single wells) for each treatment using the Grubb’s test, at most one value (single well from the six to twelve technical replicates on an individual plate) was excluded for each test if identified as a significant outlier (Alpha = 0.05). Plate-median normalized data were grouped by treatment condition and exported to Prism software (version 6, GraphPad, San Diego, CA) for statistical analysis and plotting. LIVE/DEAD, JC-1, and TMRE data were acquired in BD FACSDiva software (version 8.0.2, BD Biosciences). For the JC-1 assay, fluorescence ratios of red to green were calculated using Excel, grouped by treatment condition, and exported to Prism for statistical analysis and plotting. Seahorse data were acquired in Wave software (version 2.2.0, Agilent Technologies) and exported to Excel for processing. Outlier exclusions were applied to data points for each treatment to identify within-plate (single wells) and between-plate variations using the Grubb’s test, at most one value (single well from the six to twelve technical replicates on an individual plate or the averaged value from one biological replicate) was excluded if identified as a significant outlier (Alpha = 0.05). Total protein normalized data were grouped by treatment condition and exported to Prism for statistical analysis and plotting.

Data were analyzed statistically using one-way (ATP, Alamar, LDH, JC-1, TMRE, and LIVE/DEAD assays) or two-way (Seahorse XF Cell Mito Stress Test assay) analysis of variance (ANOVA) and the Dunnett’s or Tukey’s multiple comparisons post-hoc test using Prism software (Graph Pad Software, La Jolla, CA). Statistical significance was defined as *p* < 0.05.

## Results

### Plasma-Relevant Concentrations of DTG and EFV Impair Mitochondrial ATP Production in HeLa Cells.

**Figure 1:**
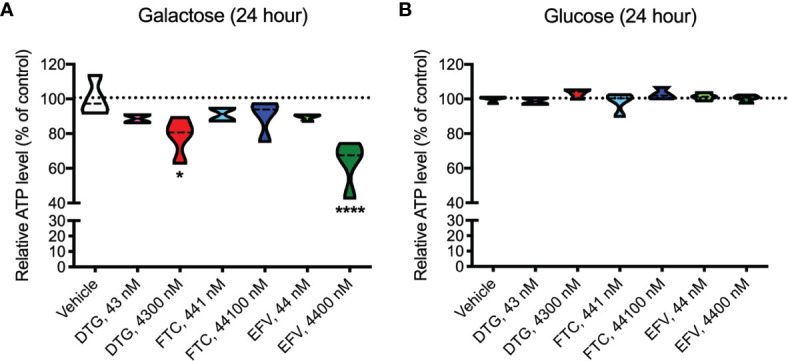
Lower mitochondrial ATP generation in HeLa cells treated with DTG and EFV. HeLa cells were incubated for 24 hours with DTG, FTC, or EFV at the stated concentrations in the absence **(A)** or presence **(B)** of glucose. The level of ATP is given as a percentage of vehicle-treated (0.1% DMSO) cells. Statistically significant compared to vehicle (p < 0.05*, 0.0001****). n=4.

ATP was measured in HeLa cells after incubation with DTG, FTC, and EFV at CSF-relevant concentrations and a 100x higher concentration to approximate plasma-relevant concentrations for DTG and EFV (**Table 1**). Both glucose-containing and glucose-free (supplemented with galactose) media were used, the latter to ensure the highly glycolytic HeLa cells use mitochondrial oxidative phosphorylation. We found that plasma-relevant concentrations of DTG (4300 nM) and EFV (4400 nM) decreased ATP after 24-hours in glucose-free (**Figure 1A**), but not in glucose-containing (**Figure 1B**) media, suggesting that similar to EFV, DTG affects mitochondria.

**Table 1:** Median ARV drug concentrations measured in the CSF and plasma of treated HIV patients.

ARV drug	CSF		Plasma		Reference
	ng/mL	nM	ng/mL	nM	
DTG	18	43	3360	8000	(20)
FTC	109	441	254	1027	(31)
EFV	13.9	44	2145	6795	(31)

### Mitochondrial Membrane Potential Remains Unaltered in ARV Exposed HeLa Cells

**Figure 2:**
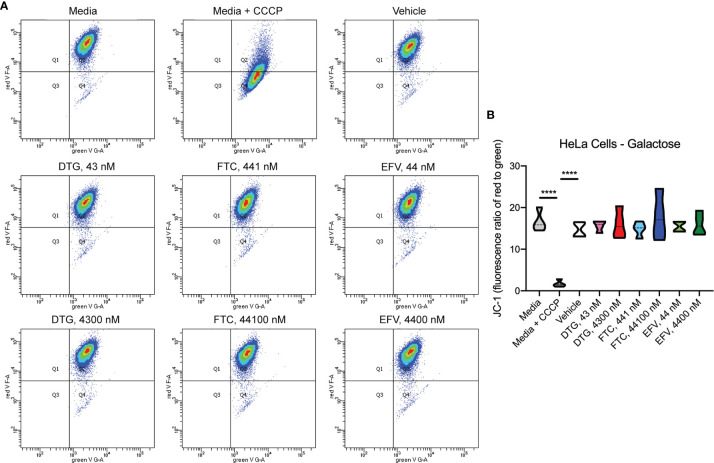
No alteration in HeLa cell mitochondrial membrane potential due to ARV treatment. HeLa cells were incubated with JC-1 dye following incubation with DTG, FTC, or EFV at the stated concentrations for 24 hours in the absence of glucose. Treatment with 20 µM CCCP for 30 min was used as a positive control for mitochondrial depolarization. **(A)** Representative flow cytometry plot showing JC-1 staining in media, and after treatment with CCCP, vehicle, and ARV drugs. **(B)** Graph showing fluorescence ratio of red to green for JC-1 staining. Statistically significant (p < 0.0001****). n=4.

To assess if mitochondrial ATP alterations upon exposure to DTG and EFV for 24-hours in glucose-free conditions coincide with mitochondrial membrane potential changes, the cells were incubated with the widely used cationic dye, JC-1. When mitochondria are well polarized, JC-1 aggregates in mitochondria fluorescing red. In mitochondria with low membrane potential, JC-1 remains in the monomeric form, which fluoresces green. No changes in mitochondrial membrane potential were observed (**Figure 2**). To confirm the JC-1 results, mitochondrial membrane potential was also assessed using the cell-permeant, cationic, red-orange fluorescent dye tetramethylrhodamine, ethyl ester (TMRE) that is readily sequestered by active mitochondria. Consistent with the JC-1 results, no changes in mitochondrial membrane potential were observed (**Supplementary Figure S1**).

### Similar to EFV, DTG and FTC Alter Mitochondrial REDOX Activity in HeLa Cells

**Figure 3:**
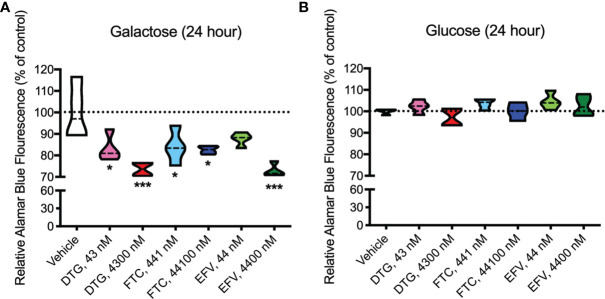
Decreased mitochondrial REDOX in HeLa cells treated with DTG, FTC, and EFV. HeLa cells were incubated for 24 hours with DTG, FTC, or EFV at the stated concentrations in the absence **(A)** or presence **(B)** of glucose. The reduction of Alamar Blue is given as a percentage of vehicle-treated (0.1% DMSO) cells. Statistically significant compared to vehicle (p < 0.05*, 0.001***). n=4.

Upon accepting electrons from mitochondrial reductases and/or diaphorase-type enzymes or from non-mitochondrial cytosolic enzymes (34), the REDOX indicator Alamar Blue changes from the oxidized, non-fluorescent, blue state to the reduced, fluorescent, pink state. These studies were conducted in the absence (**Figure 3A**) and presence (**Figure 3B**) of glucose revealing that 24-hour treatment with CSF-relevant and 100x higher concentrations of DTG (43 and 4300 nM) and FTC (441 and 44100 nM), and plasma-relevant concentrations of EFV (4400 nM) impairs dye reduction in the absence of glucose, pointing to interruption of mitochondrial electron transport. The observed effects of DTG and FTC on ATP and REDOX in HeLa cells are not due to cell death, as no increase in LDH/cell death was uncovered; however, plasma-relevant EFV concentrations (4400 nM) did increase cell death in glucose-free media (**Supplementary Figure S2**). Cell viability, assessed by flow cytometry using LIVE/DEAD fluorescence, revealed no change in the percent of cells alive confirming the LDH results (**Supplementary Figures S3**, **S4**).

### DTG and FTC Alter Cellular Metabolism in BV2 Cells in the Absence of Cell Death

**Figure 4:**
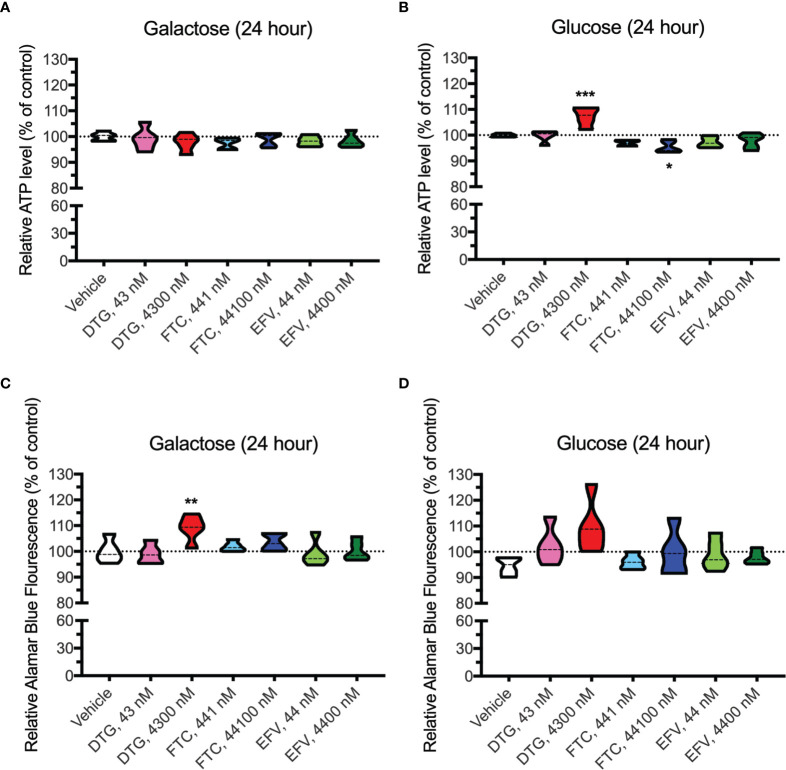
Altered glycolytic ATP levels and mitochondrial REDOX in BV2 cells treated with DTG and FTC. BV2 cells were incubated for 24 hours with DTG, FTC, or EFV at the stated concentrations in the absence **(A, C)** or presence **(B, D)** of glucose. The level of ATP **(A, B)** and reduction of Alamar Blue **(C, D)** are given as a percentage of vehicle-treated (0.1% DMSO) cells. Statistically significant compared to vehicle (p < 0.05*, 0.01**, 0.001***). n=5.

**Figure 5:**
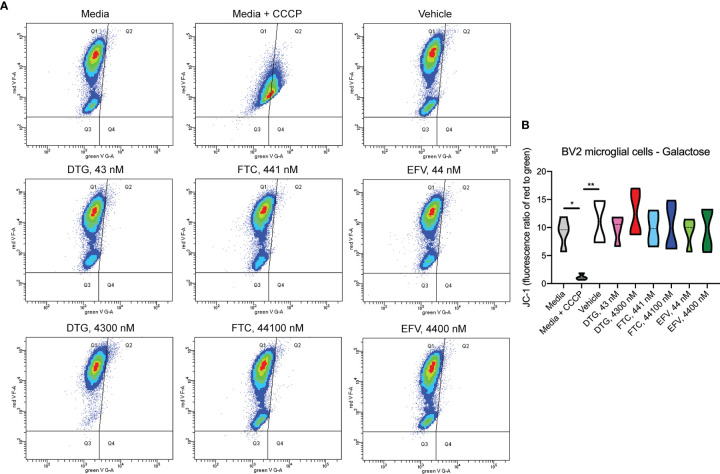
No alteration in BV2 cell mitochondrial membrane potential due to ARV treatment in the absence of glucose. BV2 cells were incubated with JC-1 dye following incubation with DTG, FTC, or EFV at the stated concentrations for 24 hours in glucose-free media. Treatment with 20 µM CCCP for 30 min was used as a positive control for mitochondrial depolarization. **(A)** Representative flow cytometry plot showing JC-1 staining in media, and after treatment with CCCP, vehicle, and ARV drugs. **(B)** Graph showing fluorescence ratio of red to green for JC-1 staining. Statistically significant (p < 0.05*, 0.01**). n=4.

**Figure 6:**
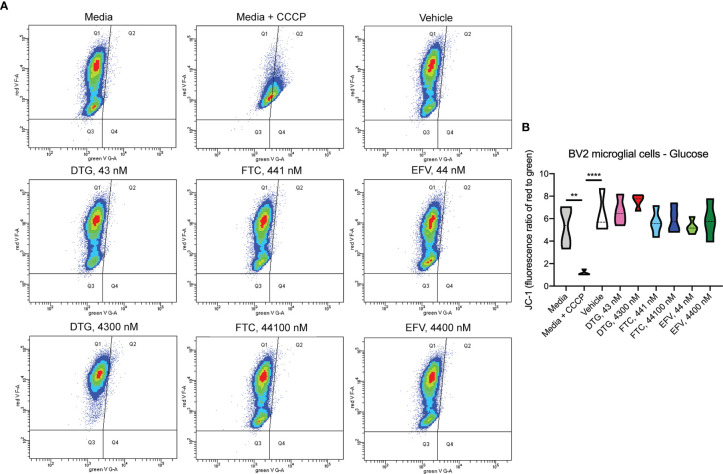
No alteration in BV2 cell mitochondrial membrane potential due to ARV treatment in the presence of glucose. BV2 cells were incubated with JC-1 dye following incubation with DTG, FTC, or EFV at the stated concentrations for 24 hours in glucose-containing media. Treatment with 20 µM CCCP for 30 min was used as a positive control for mitochondrial depolarization. **(A)** Representative flow cytometry plot showing JC-1 staining in media, and after treatment with CCCP, vehicle, and ARV drugs. **(B)** Graph showing fluorescence ratio of red to green for JC-1 staining. Statistically significant (p < 0.01**, 0.0001****). n=4.

In contrast to highly glycolytic HeLa cells, where mitochondrial alterations were uncovered in the absence of glucose, in BV2 cells, no ATP changes were uncovered in glucose-free media (**Figure 4A**). However, in glucose-containing media, plasma-relevant DTG concentrations (4300 nM) increased ATP and the 100x higher FTC concentration (44100 nM) decreased ATP (**Figure 4B**), suggesting glycolytic ATP production is altered. Interestingly, plasma-relevant DTG concentrations increased Alamar Blue fluorescence in the absence of glucose (**Figure 4C**) but not in the presence of glucose (**Figure 4D**), suggesting mitochondrial REDOX is elevated. Similar to HeLa cells, in BV2 cells the mitochondrial membrane potential remained unaltered in glucose-free (**Figure 5** and **Supplementary Figure S5**) and glucose-containing (**Figure 6** and **Supplementary Figure S6**) conditions. Of note, while the positive control (FCCP) yielded significant loss of TMRE fluorescence, characteristic of depolarization, in the HeLa cell experiments in glucose-free and in the BV2 cell experiments in glucose-containing conditions, this was not the case in the BV2 cell experiments in glucose-free conditions (despite titration of FCCP concentration). Rhodamine dye and its derivatives, such as TMRE, can be pumped out from cells by multidrug resistance proteins, and BV2 cells do express these proteins and depending on the cellular environment, retention of such dyes can be impaired in this cell line (35). Other reports of lack of spectral shifts in fluorescence of rhodamine derivatives in the presence of uncouplers have been observed (36). As such, JC-1 might be a more reliable indicator of mitochondrial membrane potential than TMRE for BV2 cells in glucose-free, galactose-containing conditions, similar to reports for other specific cell types and conditions (37). DTG, FTC, and EFV were not cytotoxic in BV2 cells as assessed by the LDH assay (**Supplementary Figure S7**) and flow cytometry LIVE/DEAD fluorescence (**Supplementary Figures S8**, **S9**).

### Bioenergetic Alterations in BV2 Cells Due to ARV Drug Exposure

**Figure 7:**
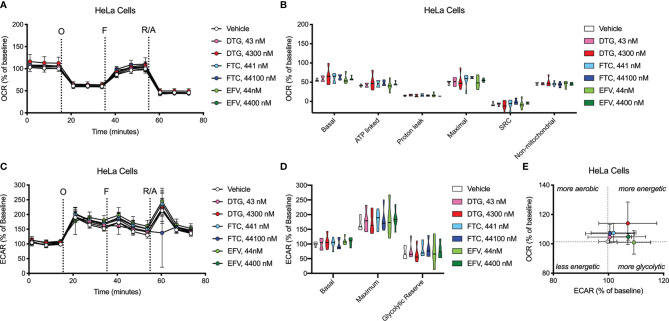
HeLa cell bioenergetics remain intact after ARV treatment. HeLa cells were incubated for 24 hours with DTG, FTC, or EFV at the stated concentrations. **(A)** Graphical representation of the relative OCR responses over time expressed as a percent response from baseline (third measurement, before oligomycin injection); sequential additions are indicated as O (the ATP synthase inhibitor oligomycin, 1 μM), F (the ATP synthesis uncoupler FCCP, 300 nM), and R/A (a mixture of the complex I and III inhibitors rotenone and antimycin A, 2 μM each). **(B)** Mitochondrial respiratory parameters calculated from the OCR shown for basal mitochondrial respiration (baseline minus R/A), ATP linked respiration (baseline minus O), proton leak (O minus R/A), maximal mitochondrial respiration (F minus R/A), and SRC (F minus baseline). **(C)** Graphical representation of the relative ECAR responses over time. **(D)** Glycolytic rate parameters calculated from ECAR shown for basal glycolysis (baseline), maximal glycolysis (O), and glycolytic reserve (O minus baseline). **(E)** Plot of baseline ECAR and OCR levels. n=5.

**Figure 8:**
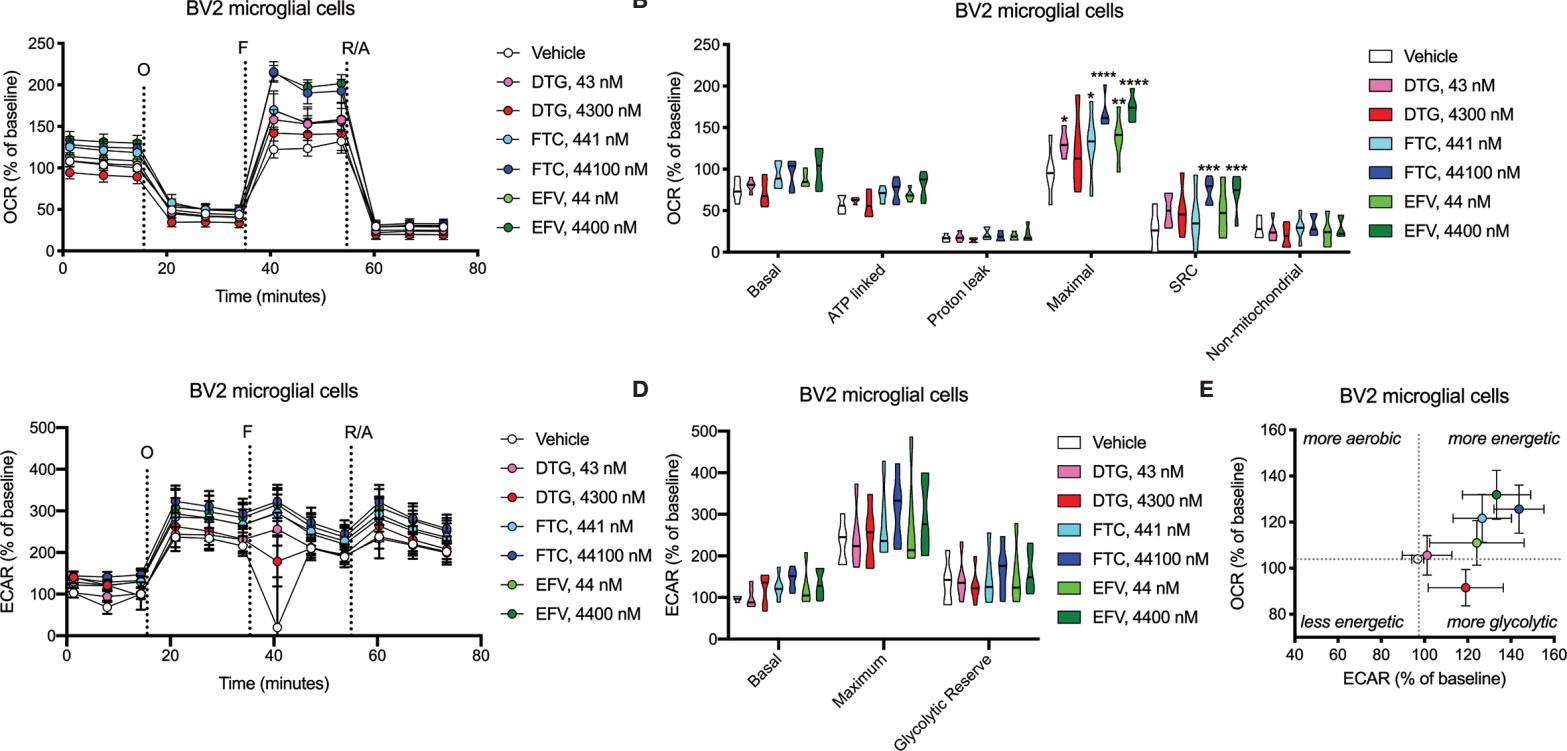
BV2 cell bioenergetics altered after ARV treatment. BV2 cells were incubated for 24 hours with DTG, FTC, or EFV at the stated concentrations. **(A)** Graphical representation of the relative OCR responses over time expressed as a percent response from baseline (third measurement, before oligomycin injection); sequential additions are indicated as O (the ATP synthase inhibitor oligomycin, 1 μM), F (the ATP synthesis uncoupler FCCP, 300 nM), and R/A (a mixture of the complex I and III inhibitors rotenone and antimycin A, 2 μM each). **(B)** Mitochondrial respiratory parameters calculated from the OCR shown for basal mitochondrial respiration (baseline minus R/A), ATP linked respiration (baseline minus O), proton leak (O minus R/A), maximal mitochondrial respiration (F minus R/A), and SRC (F minus baseline). **(C)** Graphical representation of the relative ECAR responses over time. **(D)** Glycolytic rate parameters calculated from ECAR shown for basal glycolysis (baseline), maximal glycolysis (O), and glycolytic reserve (O minus baseline). **(E)** Plot of baseline ECAR and OCR levels. (Statistically significant compared to vehicle (p < 0.05*, 0.01**, 0.001***, 0.0001****). n=5.

OCR (mitochondrial respiration) and ECAR (glycolysis) were measured after 24-hours of DTG, FTC, and EFV exposure in HeLa and BV2 cells using the Seahorse XFe96 Analyzer. Sequential additions of an ATP synthase inhibitor (O), ATP synthesis uncoupler (F), and mixture of complex I and III inhibitors (R/A) allowed determination of basal mitochondrial respiration, ATP production-linked rate, proton leakage, maximal mitochondrial respiration, spare respiratory capacity (SRC), and non-mitochondrial respiration. No significant alterations were uncovered in HeLa cells (**Figure 7**); however, the cells appear to shift towards becoming more glycolytic and less aerobic, particularly for both EFV and high DTG concentrations (**Figure 7E**). In contrast, in BV2 cells, maximal mitochondrial respiration was increased upon exposure to CSF-relevant DTG, FTC, and EFV, and the 100x higher FTC and EFV concentrations (**Figures 8A, B**). Further, mitochondrial SRC was elevated following exposure to the 100x higher FTC and EFV concentrations (**Figure 8B**). While no significant ECAR changes were uncovered (**Figures 8C, D**), the cells appear to become more energetic overall (more aerobic and glycolytic), particularly for both FTC and EFV concentrations (**Figure 8E**). The cell energy phenotype of HeLa and BV2 cells was determined by plotting ECAR (glycolysis) as a function of OCR (mitochondrial respiration) revealing that under baseline conditions both cell lines utilize both energy pathways; however, as compared to HeLa cells, we found that BV2 cells are less glycolytic and more aerobic (**Supplementary Figure S10**).

## Discussion

Neurocognitive abnormalities continue to occur in HIV-infected individuals, despite the widespread use of cART (3–5). Neuropsychiatric symptoms, including depression and anxiety disorders, mood and sleep disorders, and suicidal ideation, are common in people living with HIV and may be associated with specific ARV drugs (1, 38–40). Fifty percent of those taking EFV experience neuropsychiatric adverse effects, including vivid dreams, dizziness, balance problems, unsteadiness, light-headedness, and suicidal ideation (9). The precise mechanisms of ARV toxicity are not fully understood, but in the case of EFV, we (6, 7), and others (8–10) have found effects on neuronal mitochondria. While EFV use is decreasing, there are other CNS-penetrant ARV drugs that are currently recommended, including DTG and FTC, that we studied herein.

DTG, a second-generation integrase inhibitor, is recommended as one of the preferred options for first-line HIV treatment in European and United States treatment guidelines. Recent reports from clinical practice of neuropsychiatric adverse events with integrase inhibitors (39, 41), and DTG in particular, have highlighted a need to understand the off-target effects of these medications and which individuals are at greatest risk, particularly as use of DTG is increased in patients. In fact, adverse CNS side effects of DTG are occurring more frequently with everyday use than clinical trials had predicted. The most frequent manifestations reported as leading to discontinuation were insomnia, dizziness, headache, anxiety, and depression (39, 41). These adverse effects are more likely to occur in women, people over 60, and those starting abacavir at the same time (41). While all medications have potential side effects, it is important that these do not initiate or worsen any of the problems that HIV infection has caused in the brain or elsewhere in the body. DTG is CNS penetrant, and known to reach effective concentrations in the CSF (20). As DTG is now a commonly used ARV option in naïve and pretreated patients, further research on its safety and neurotoxicity are needed. FTC is a NRTI with a relatively good tolerability and safety profile. However, nervous system side effects have been reported in FTC-containing cART, including headache, paresthesia, confusion, irritability, depression, and insomnia. While FTC and DTG (together with tenofovir – TDF or TAF, both non-CNS penetrant) combination is one of the recommended first line treatments for HIV, these drugs were studied individually to uncover if these drugs affect mitochondrial functions. Here, we used the glucose-galactose assay to investigate potential mitotoxic effects of DTG, FTC, and EFV in highly glycolytic HeLa cells, and upon identification of potential mitotoxicity of DTG and FTC, similar to EFV, using this screen, who chose a well-used and characterized microglial cell line, the BV2 cell line for additional experiments. We demonstrate that DTG, FTC, and EFV interfere with cellular metabolism in a cell-type specific manner, decreasing mitochondrial ATP (DTG and EFV) and mitochondrial REDOX (DTG, FTC, and EFV) in HeLa cells, but increasing glycolytic ATP (DTG) and mitochondrial REDOX (DTG) in BV2 cells. FTC was found to decrease glycolytic ATP in BV2 cells. Further, we discovered that these ARV drugs (DTG, FTC, and EFV) increased maximal mitochondrial respiration in BV2 cells.

Growing cell lines in the absence and presence of glucose in parallel to detect mitochondrial toxins is becoming more common in drug development screening activities (28–30, 42). For such glucose-galactose assays, highly glycolytic cells that are resistant to mitochondrial toxins under typical high-glucose culture conditions are typically used. Indeed, in HeLa cells, this assay uncovered reduced ATP only in glucose-free media upon ARV exposure, thus resulting from mitochondrial dysfunction. Plasma-relevant DTG concentrations caused an ~20% drop in mitochondrial ATP (**Figure 1**) in the absence of cell death, suggesting less essential ATP-consuming processes may be inhibited to preserve those more critical for cell survival. Studies show energy spared by reducing protein synthesis (25-30% of the cells ATP) can be allocated to more critical cell functions involved in osmotic and ionic homeostasis (43). Thus, future studies could interrogate the hierarchy of ATP-consuming processes in the context of ARV exposure to further characterize how the cellular metabolism is altered.

Despite ARV-induced decreases in mitochondrial ATP generation, mitochondrial membrane potential was unaltered (**Figure 2** and **Supplementary Figure S1**). The mitochondrial electrochemical gradient, which drives mitochondrial ATP synthesis, is comprised of the mitochondrial membrane potential and pH (proton) gradient (37). Changes in the mitochondrial membrane potential do not always mirror alterations in the mitochondrial pH, since each of these contribute independently to the proton-motive force. Here, mitochondrial membrane potential was measured using JC-1, a cationic dye that measures the charge gradient across the inner mitochondrial membrane but cannot specifically measure the mitochondrial proton gradient or be used to make direct inferences regarding respiratory status. Similarly, experiments using another cationic dye TMRE, which accumulates in active mitochondria due to their relative negative charge, shares these same limitations. Although most studies focus on mitochondrial membrane potential as it is easily measured, previous studies on HIV Tat-induced hyperpolarization of the mitochondrial membrane was not associated with increased ATP as expected, but did coincide with decreased mitochondrial pH (thus decreasing ATP generating capacity) suggesting increased cytosolic calcium, not protonic charges, were responsible for the hyperpolarization (44–46). Of note, mitochondrial membrane potential and proton gradient can be maintained *via* ATP synthase reversal, which depletes ATP (47). Thus, future experiments could interrogate the role of the mitochondrial proton gradient and/or calcium homeostasis, as well as ATP synthase reversal, in ARV-induced mitotoxicity.

Concurrent with reduced mitochondrial ATP, CSF-relevant and the 100x higher concentrations of FTC, the CSF- and plasma-relevant concentrations of DTG, and the plasma-relevant concentrations of EFV, impaired mitochondrial REDOX in HeLa cells in glucose-free conditions (**Figure 3**), attributing the loss of Alamar Blue reduction mainly to mitochondrial enzymes and electron carriers. Again, while mitochondrial membrane potential remained unaltered (**Figure 2** and **Supplementary Figure S1**), impairment of the mitochondrial membrane potential or the mitochondrial pH gradient can facilitate mitochondrial redox dysfunction. Heart studies have shown that the mitochondrial pH gradient restricts electron flow, controls superoxide generation, and results in a more reduced environment through imposed proton backpressure, while impairment of the mitochondrial pH gradient leads to a more oxidized environment (48). Thus, ARV-induced REDOX dysfunction (more oxidized Alamar Blue) may correlate with mitochondrial pH gradient impairment in the absence of mitochondrial membrane potential changes. While no changes in ECAR (glycolysis) or OCR (mitochondrial respiration) were uncovered in the HeLa cells (**Figure 7**), these experiments were performed with glucose present potentially masking the ARV-induced mitotoxic effects. Of note, the bioenergetic profile (**Figure 7E**) suggests that overall HeLa cells become more reliant on glycolysis when treated with DTG (plasma-relevant) and EFV (plasma- and CSF-relevant).

Dysfunctional microglia may contribute to HIV-associated cognitive disorder development, as well as neuropsychiatric adverse effects of ARV drugs (23, 24). While microglia rely on both glycolytic and mitochondrial metabolism depending on their activation state, microglial activation has been suggested to be associated with a metabolic switch in favor of glycolysis and decreased mitochondrial oxidative phosphorylation (25, 26). CSF-relevant DTG, FTC, and EFV concentrations did not affect ATP or REDOX in BV2 cells; however, these effects were only studied following 24-hour exposure to assess the effects of one dose equivalent (DTG, FTC, and EFV are usually taken once daily, while DTG can be taken twice daily). Patients would take these drugs daily throughout their lives, so future experiments should assess chronic exposure to CSF-relevant drug levels. Of note, CSF-relevant DTG, FTC, and EFV concentrations elevated maximal mitochondrial respiration in BV2 cells (**Figure 8**). Overall, the bioenergetic profile suggests BV2 cells become more energetic upon FTC and EFV exposure (**Figure 8E**). These findings suggest ARV exposure might metabolically reprogram the microglia towards a resting state (increased mitochondrial respiration), which could be beneficial during chronic neuroinflammation but might also inhibit acute neuroinflammatory responses.

While CSF-relevant concentrations of the ARV drugs may be applicable to studies on microglia, we note that a recent study in rhesus monkeys indicates that ARV drugs are found at concentrations ranging from 13 to 1,150-fold higher in brain tissue than in CSF (49). Thus, our higher concentrations may be quite appropriate for the brain, and the cells within the brain such as microglia. In addition, such higher concentrations are consistent with acute toxicity screens, including previous acute treatment studies on metabolic effects using relatively high ARV drug concentrations to uncover metabolic effects as discussed below. We found that these higher, plasma-relevant concentrations of DTG increased glycolytic ATP generation, while also elevating mitochondrial reducing capacity in BV2 microglial cells (**Figure 4**). While no changes in ECAR (glycolysis) or OCR (mitochondrial respiration) were uncovered upon exposure to plasma-relevant concentrations of DTG (**Figure 8**), the bioenergetic profile suggests a switch towards a more glycolytic phenotype consistent with the ATP data. Evidence suggests metabolic reprogramming can affect microglia-derived inflammation (25), thus a shift towards a more glycolytic phenotype upon DTG exposure might induce microglial activation. The 100x higher concentrations of FTC and EFV increased both mitochondrial maximal respiration and SRC in BV2 cells (**Figure 8**). This correlates with reduced glycolytic ATP uncovered with FTC exposure (**Figure 4**), suggesting these drugs might maintain microglia in a resting state or inhibit their activation. Based on these findings, as mentioned above we anticipate that chronic exposure to CSF-relevant concentrations would alter ATP and REDOX in BV2 cells.

EFV alters the mitochondrial membrane potential in various cell-types; however, much higher EFV concentrations were used than studied here. EFV (10,000 nM, 6 hours) lowered the mitochondrial membrane potential in SH-SY5Y and U-251MG cells (18). While EFV decreased ATP in SH-SY5Y cells (25,000 nM, 24 hours), ATP levels were increased in U-251MG cells (10,000 nM, 24 hours), and these findings were confirmed in primary rat cortical neurons and astrocytes, which were more sensitive than the immortal lines (18). EFV reduced ATP in rat striatal primary neurons (12,500 nM, 2 hours) (6) and neural stem cells (5000 nM, 24 hours) (17). High EFV concentrations decrease the mitochondrial membrane potential in Hep3B cells (25,000 nM, 4 hours), and primary human hepatocytes were more sensitive than immortal lines (11). Primary mouse hepatocytes exhibit reduced ATP following EFV exposure (40,000 nM, 3 hours) (50). In CEM cells, EFV (12,700 nM, 24 hours) reduced the mitochondrial membrane potential and basal mitochondrial respiration (51). EFV (10,000 nM) acutely decreased oxygen consumption in SH-SY5Y and U-251MG (18), as well as Hep3B cells (12). Our previous studies in rat synaptosomes revealed that EFV (6250 nM, 2 hours) decreased maximal mitochondrial respiration and ATP levels (7). Although future work could study higher ARV concentrations, we believe studies using primary immune cells, including microglia, but also peripheral immune cells, may uncover more drastic metabolic effects of CSF- or plasma-relevant ARV concentrations.

Few reports of FTC and DTG metabolic effects exist, and much of this work has focused on combined treatment making it difficult to ascribe effects to a particular drug. FTC (10,000 nM) acutely reduced Hep3B cellular oxygen consumption (52). Our previous studies revealed FTC (25,000 nM, 2 hours) reduced maximal mitochondrial respiration in rat striatal synaptosomes (7). FTC (peak plasma concentration, 48 hours) did not alter mitochondrial membrane potential in CEM cells (51). CD4^+^ T cells from HIV-negative healthy individuals were exposed to DTG (9538 nM) or FTC (4045 nM) *in vitro* for 3 days, and while FTC did not alter mitochondrial respiration, DTG decreased basal and maximal respiration, with no effect on glycolysis (53). While the DTG concentration was higher than studied here in BV2 cells (4300 nM) perhaps the effects of DTG on mitochondrial function in peripheral immune cells are different than those on CNS immune cells.

These findings reveal that physiologically relevant concentrations of DTG and FTC alter ATP metabolism, mitochondrial REDOX activity, and cellular bioenergetics in the absence of cell death in a cell-type specific manner. Thus, cellular metabolic alterations may, at least in part, explain some of the mechanisms underlying discontinuation of certain ART regimens due to peripheral and central adverse effects and likely contribute to microglial metabolic alterations in the HIV-infected brain.

## Data Availability Statement

The original contributions presented in the study are included in the article/**Supplementary Material**. Further inquiries can be directed to the corresponding author.

## Author Contributions

KS and JG designed the experiments. JG, NR, and BL completed the ATP, Alamar, and LDH assays. JG and CS completed the flow cytometry experiments. JM completed the bioenergetic experiments. KS and HF interpreted the data and wrote the manuscript. All authors contributed to the article and approved the submitted version.

## Funding

This work was supported by National Institutes of Health under the award number P30 MH062261.

## Conflict of Interest

The authors declare that the research was conducted in the absence of any commercial or financial relationships that could be construed as a potential conflict of interest.

## Supplementary Material

Supplementary Figure 1 No alteration in HeLa cell mitochondrial membrane potential due to ARV treatment. HeLa cells were incubated with DTG, FTC, or EFV at the stated concentrations for 24 hours in the absence of glucose followed by incubation with TMRE dye. Treatment with 20 µM FCCP for 30 min was used as a positive control for mitochondrial depolarization. Statistically significant (p < 0.0001****). n=4.

Supplementary Figure 2 No HeLa cell death due to treatment with DTG and FTC. HeLa cells were incubated for 24 hours with DTG, FTC, or EFV at the stated concentrations in the absence **(A)** or presence **(B)** of glucose. Cell death measured using the LDH assay is given as a percentage of cytotoxicity. Statistically significant compared to vehicle (p < 0.05*). n=4.

Supplementary Figure 3 HeLa cell viability unaffected by ARV treatment. HeLa cells were incubated for 24 hours with DTG, FTC, or EFV at the stated concentrations in glucose-free media. Cell viability was determined using the LIVE/DEAD assay for flow cytometry and reported as the percent of viable (live) cells. n=4.

Supplementary Figure 4 HeLa cell viability unaffected by ARV treatment. HeLa cells were incubated for 24 hours with DTG, FTC, or EFV at the stated concentrations in glucose-containing media. Cell viability was determined using the LIVE/DEAD assay for flow cytometry and reported as the percent of viable (live) cells. n=4.

Supplementary Figure 5 No alteration in BV2 cell mitochondrial membrane potential due to ARV treatment. BV2 cells were incubated with DTG, FTC, or EFV at the stated concentrations for 24 hours in the absence of glucose followed by incubation with TMRE dye. Treatment with 10, 20, and 40 μM FCCP for 30 min was used as a positive control for mitochondrial depolarization. n=4.

Supplementary Figure 6 No alteration in BV2 cell mitochondrial membrane potential due to ARV treatment. BV2 cells were incubated with DTG, FTC, or EFV at the stated concentrations for 24 hours in the presence of glucose followed by incubation with TMRE dye. Treatment with 10 mM FCCP for 30 min was used as a positive control for mitochondrial depolarization. Statistically significant (p < 0.001***, 0.0001****). n=4.

Supplementary Figure 7 No BV2 microglial cell death due to ARV treatment. BV2 cells were incubated for 24 hours with DTG, FTC, or EFV at the stated concentrations in the absence **(A)** or presence **(B)** of glucose. Cell death measured using the LDH assay is given as a percentage of cytotoxicity. n=5.

Supplementary Figure 8 BV2 cell viability unaltered by ARV treatment in the absence of glucose. BV2 cells were incubated for 24 hours with DTG, FTC, or EFV at the stated concentrations in glucose-free media. Cell viability was determined using the LIVE/DEAD assay for flow cytometry and reported as the percent of viable (live) cells. n=4.

Supplementary Figure 9 BV2 cell viability unaltered by ARV treatment in the presence of glucose. BV2 cells were incubated for 24 hours with DTG, FTC, or EFV at the stated concentrations in glucose-containing media. Cell viability was determined using the LIVE/DEAD assay for flow cytometry and reported as the percent of viable (live) cells. n=4.

Supplementary Figure 10 Cell Energy Phenotype. Plot of baseline ECAR (glycolysis) and OCR (mitochondrial respiration) levels for HeLa (white dot) and BV2 (grey dot) cells. n=5.
