# Supplementary figures and images for: Physiologically Relevant Concentrations of Dolutegravir, Emtricitabine, and Efavirenz Induce Distinct Metabolic Alterations in HeLa Epithelial and BV2 Microglial Cells
Source: Front Immunol. 2021 May 20;12:639378. doi: 10.3389/fimmu.2021.639378 (PMC8173175; doi:10.3389/fimmu.2021.639378)

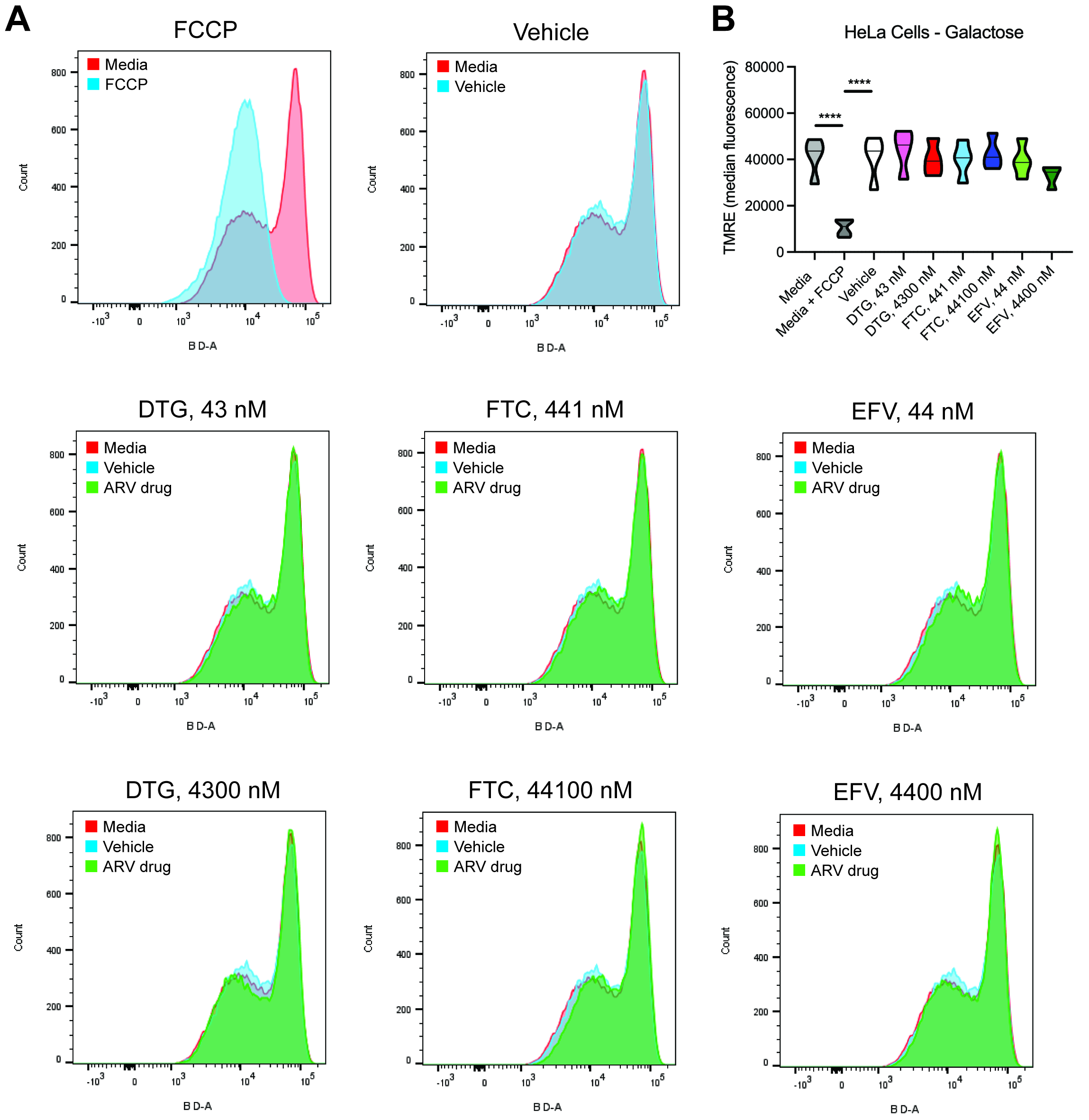

Supplement: Supplementary Figure 1 — No alteration in HeLa cell mitochondrial membrane potential due to ARV treatment. HeLa cells were incubated with DTG, FTC, or EFV at the stated concentrations for 24 hours in the absence of glucose followed by incubation with TMRE dye. Treatment with 20 µM FCCP for 30 min was used as a positive control for mitochondrial depolarization. Statistically significant (p < 0.0001****). n=4. [file Image_1.tif]

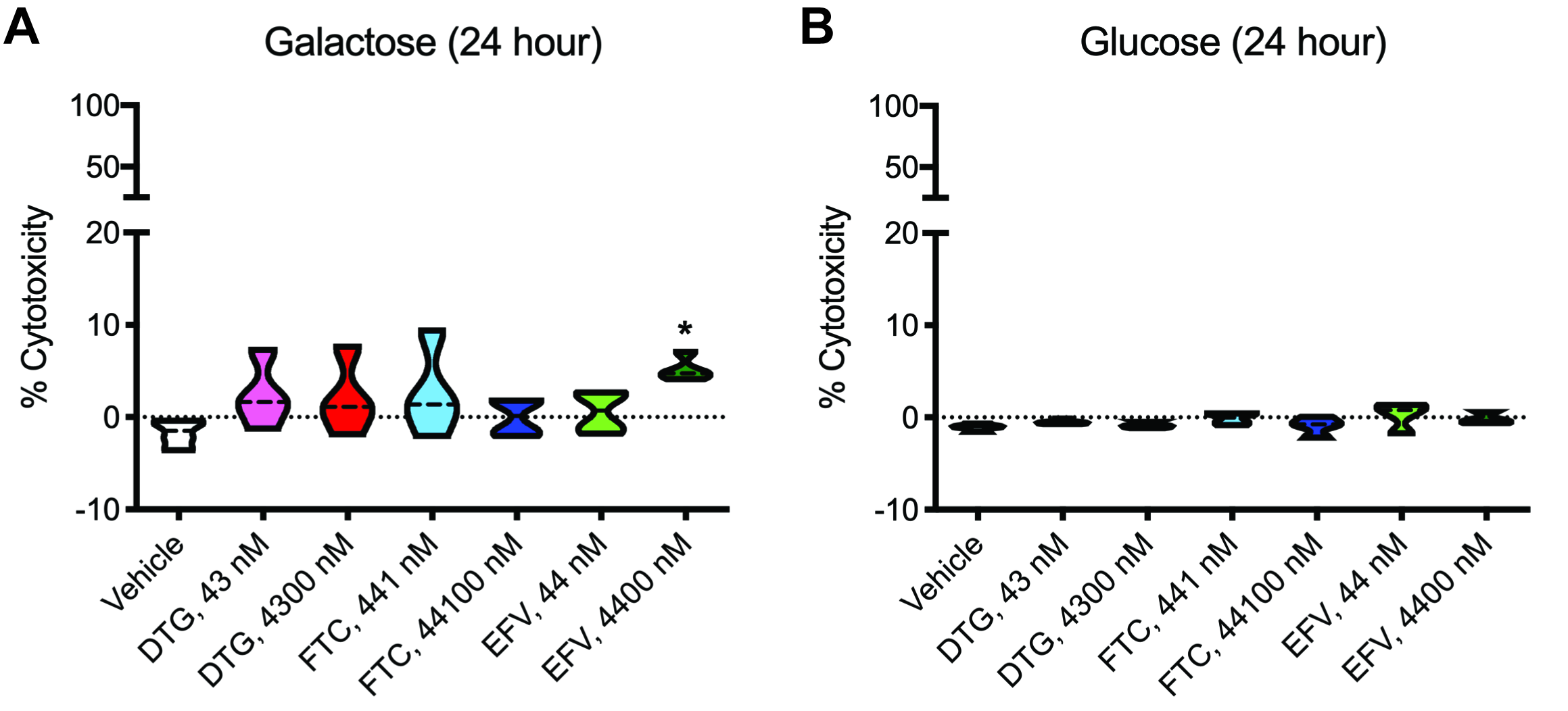

Supplement: Supplementary Figure 2 — No HeLa cell death due to treatment with DTG and FTC. HeLa cells were incubated for 24 hours with DTG, FTC, or EFV at the stated concentrations in the absence (A) or presence (B) of glucose. Cell death measured using the LDH assay is given as a percentage of cytotoxicity. Statistically significant compared to vehicle (p < 0.05*). n=4. [file Image_2.tif]

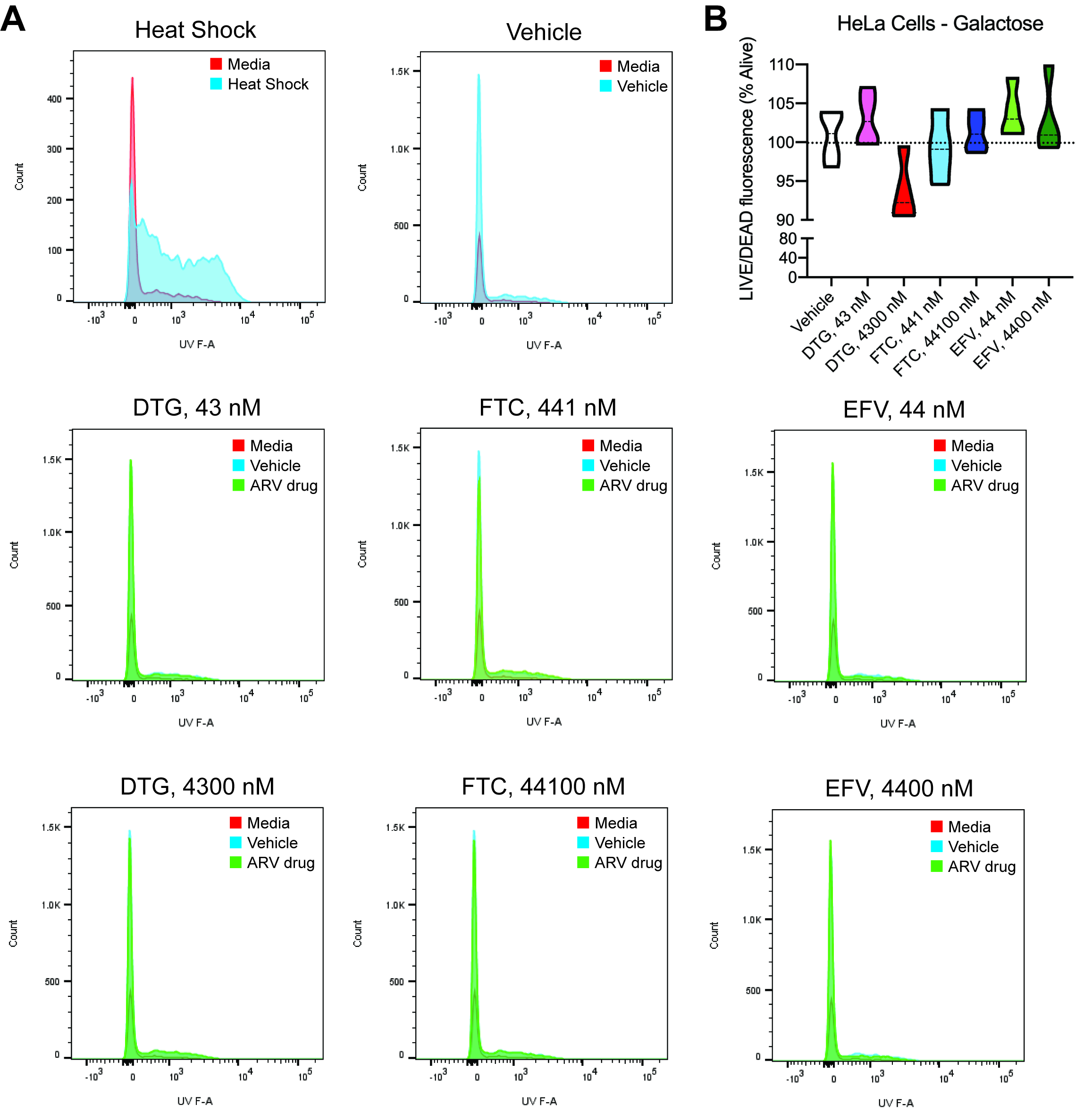

Supplement: Supplementary Figure 3 — HeLa cell viability unaffected by ARV treatment. HeLa cells were incubated for 24 hours with DTG, FTC, or EFV at the stated concentrations in glucose-free media. Cell viability was determined using the LIVE/DEAD assay for flow cytometry and reported as the percent of viable (live) cells. n=4. [file Image_3.tif]

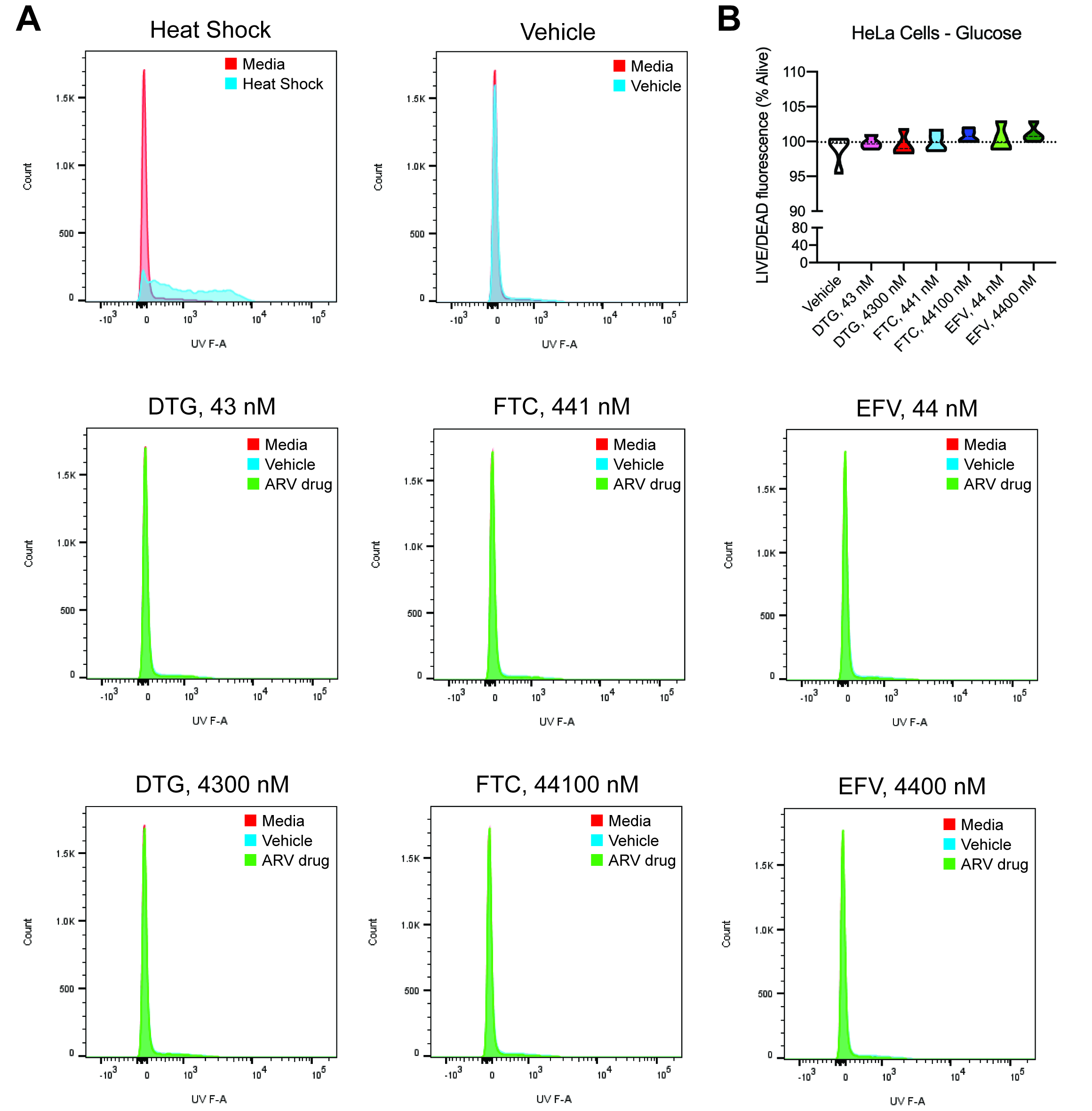

Supplement: Supplementary Figure 4 — HeLa cell viability unaffected by ARV treatment. HeLa cells were incubated for 24 hours with DTG, FTC, or EFV at the stated concentrations in glucose-containing media. Cell viability was determined using the LIVE/DEAD assay for flow cytometry and reported as the percent of viable (live) cells. n=4. [file Image_4.tif]

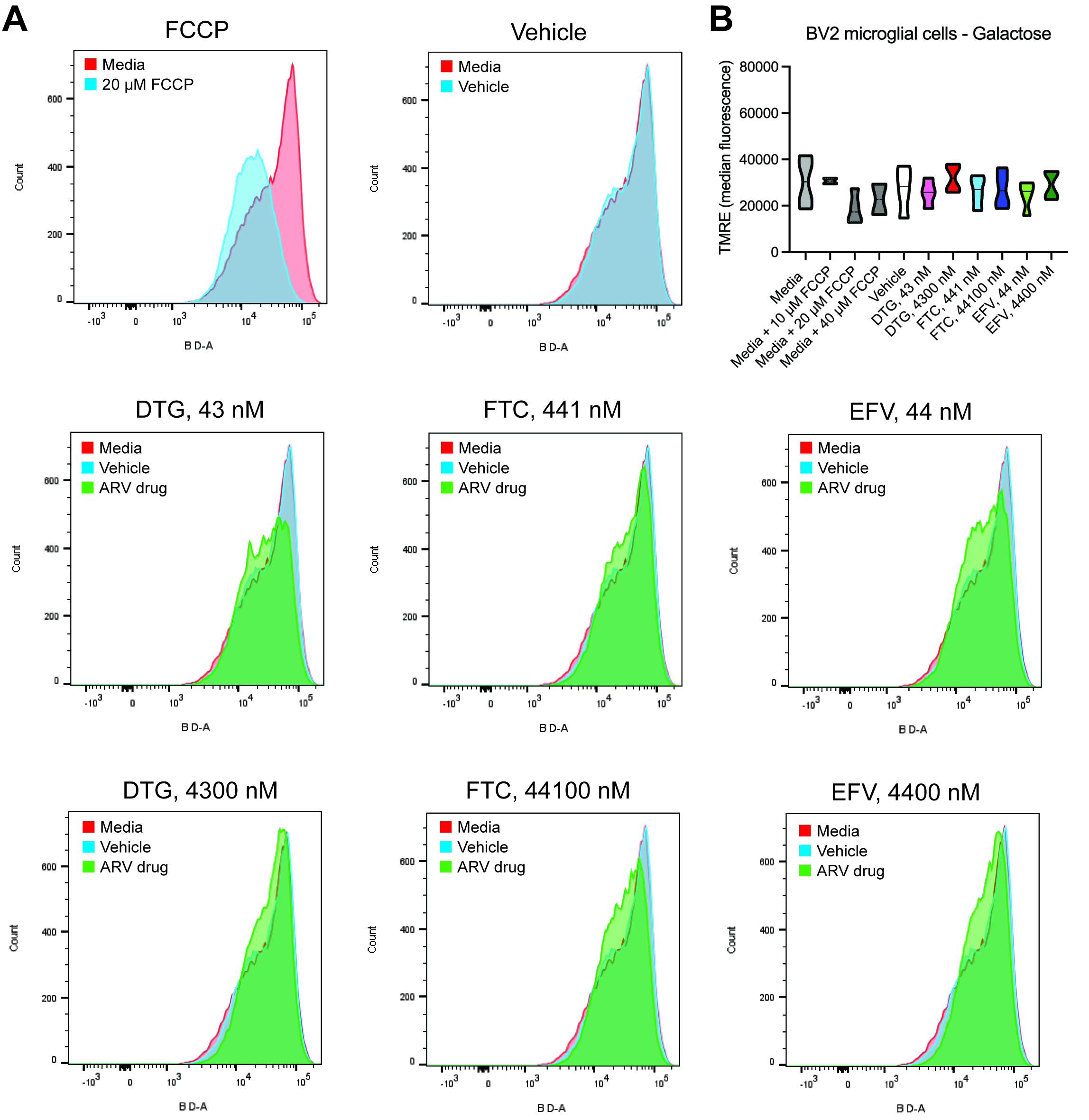

Supplement: Supplementary Figure 5 — No alteration in BV2 cell mitochondrial membrane potential due to ARV treatment. BV2 cells were incubated with DTG, FTC, or EFV at the stated concentrations for 24 hours in the absence of glucose followed by incubation with TMRE dye. Treatment with 10, 20, and 40 μM FCCP for 30 min was used as a positive control for mitochondrial depolarization. n=4. [file Image_5.tif]

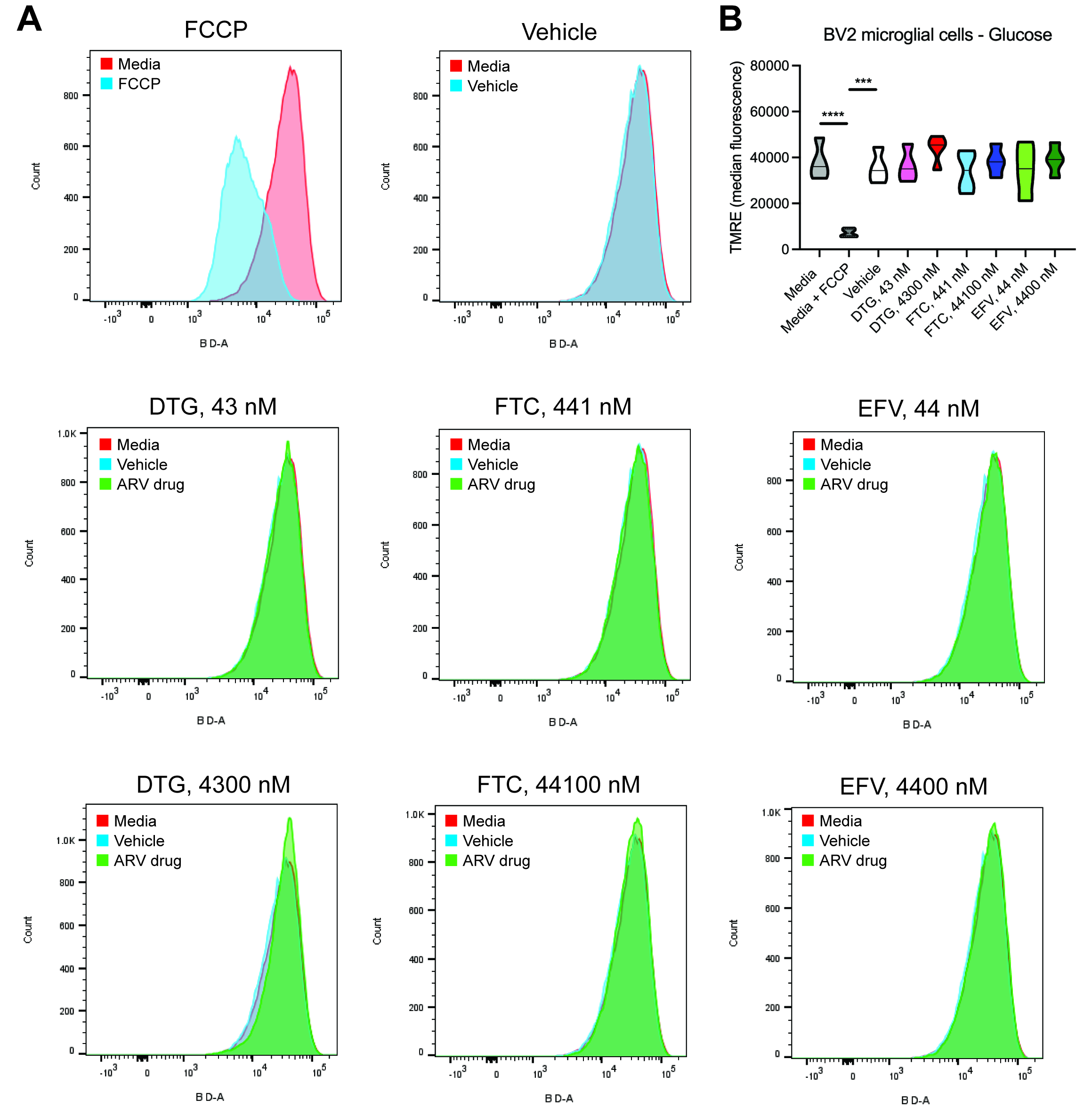

Supplement: Supplementary Figure 6 — No alteration in BV2 cell mitochondrial membrane potential due to ARV treatment. BV2 cells were incubated with DTG, FTC, or EFV at the stated concentrations for 24 hours in the presence of glucose followed by incubation with TMRE dye. Treatment with 10 mM FCCP for 30 min was used as a positive control for mitochondrial depolarization. Statistically significant (p < 0.001***, 0.0001****). n=4. [file Image_6.tif]

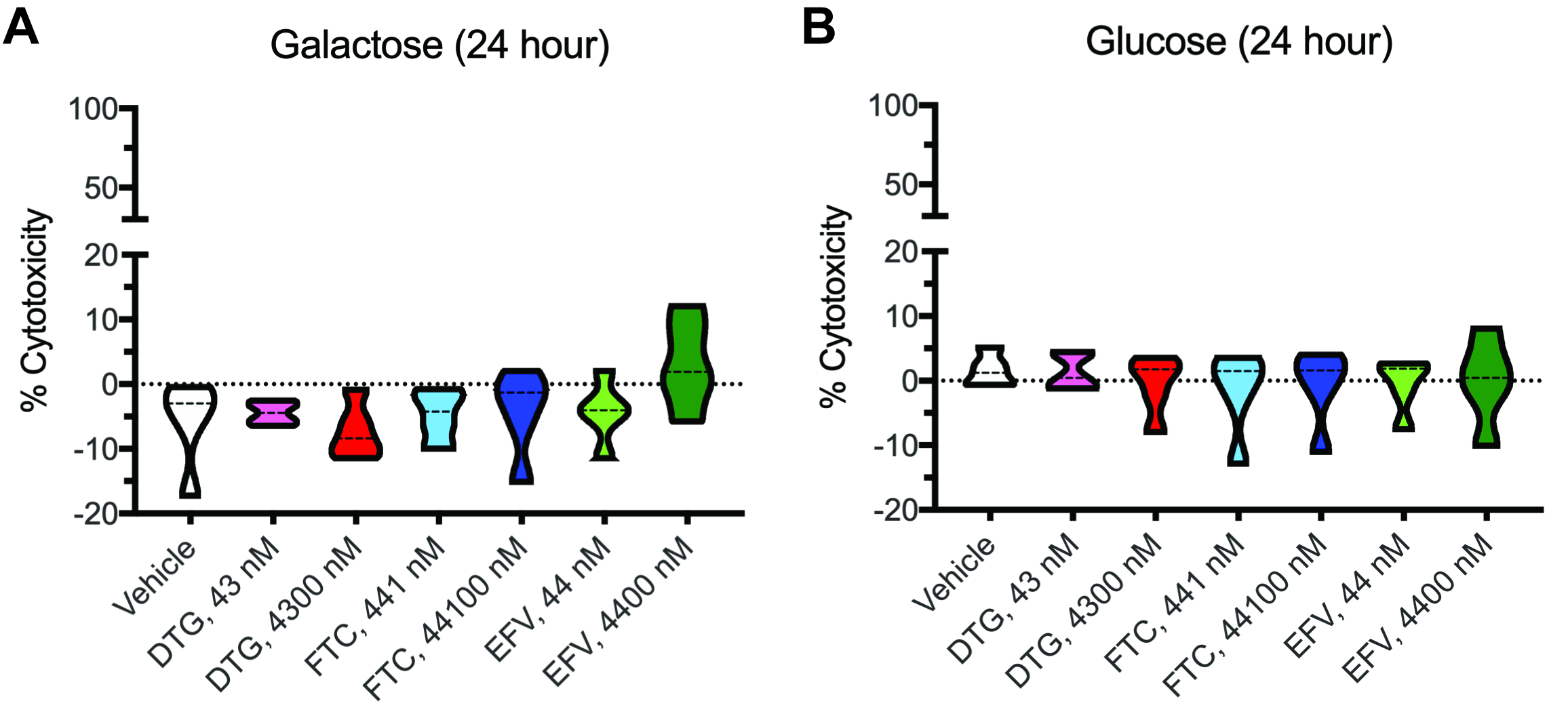

Supplement: Supplementary Figure 7 — No BV2 microglial cell death due to ARV treatment. BV2 cells were incubated for 24 hours with DTG, FTC, or EFV at the stated concentrations in the absence (A) or presence (B) of glucose. Cell death measured using the LDH assay is given as a percentage of cytotoxicity. n=5. [file Image_7.tif]

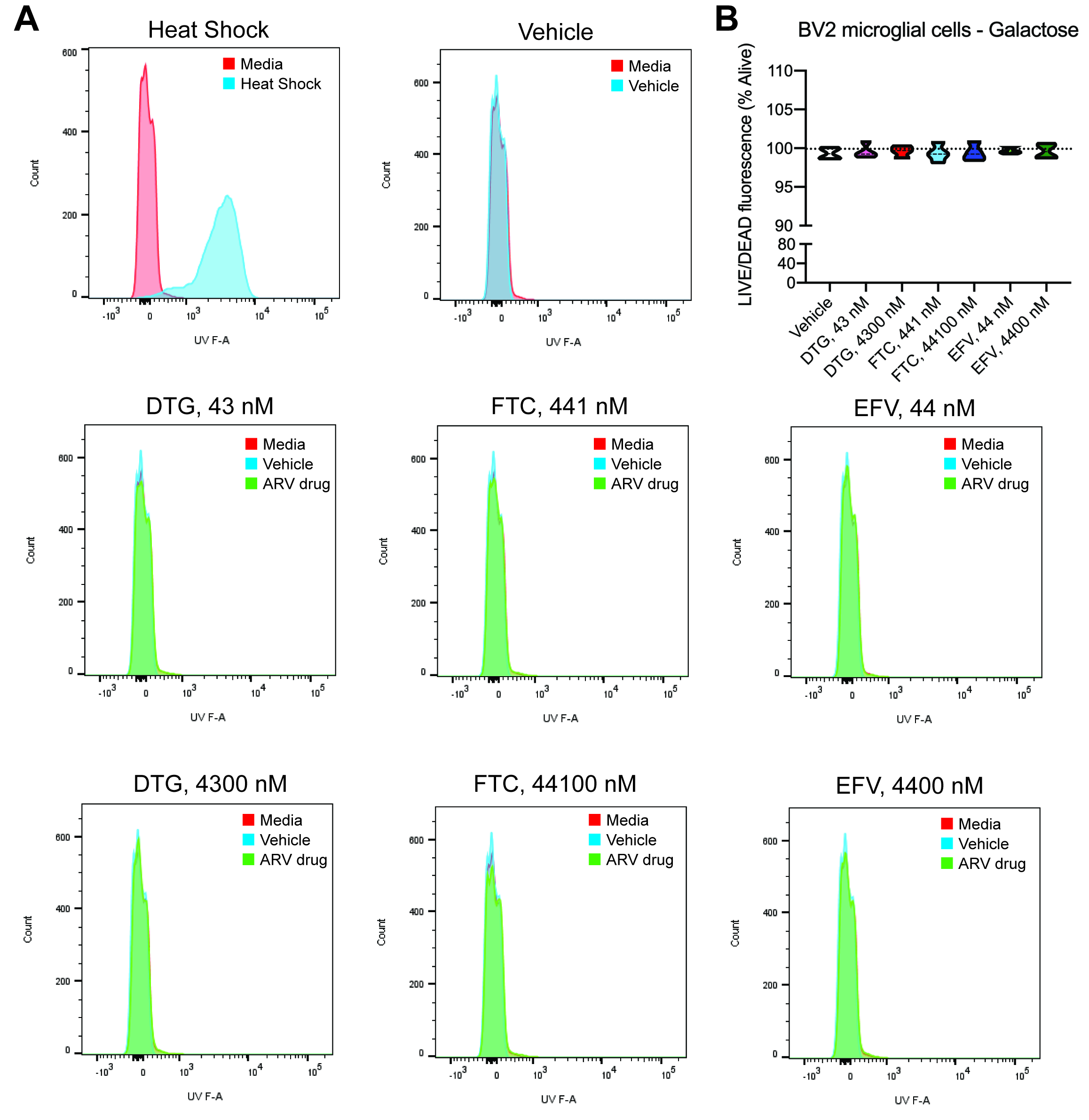

Supplement: Supplementary Figure 8 — BV2 cell viability unaltered by ARV treatment in the absence of glucose. BV2 cells were incubated for 24 hours with DTG, FTC, or EFV at the stated concentrations in glucose-free media. Cell viability was determined using the LIVE/DEAD assay for flow cytometry and reported as the percent of viable (live) cells. n=4. [file Image_8.tif]

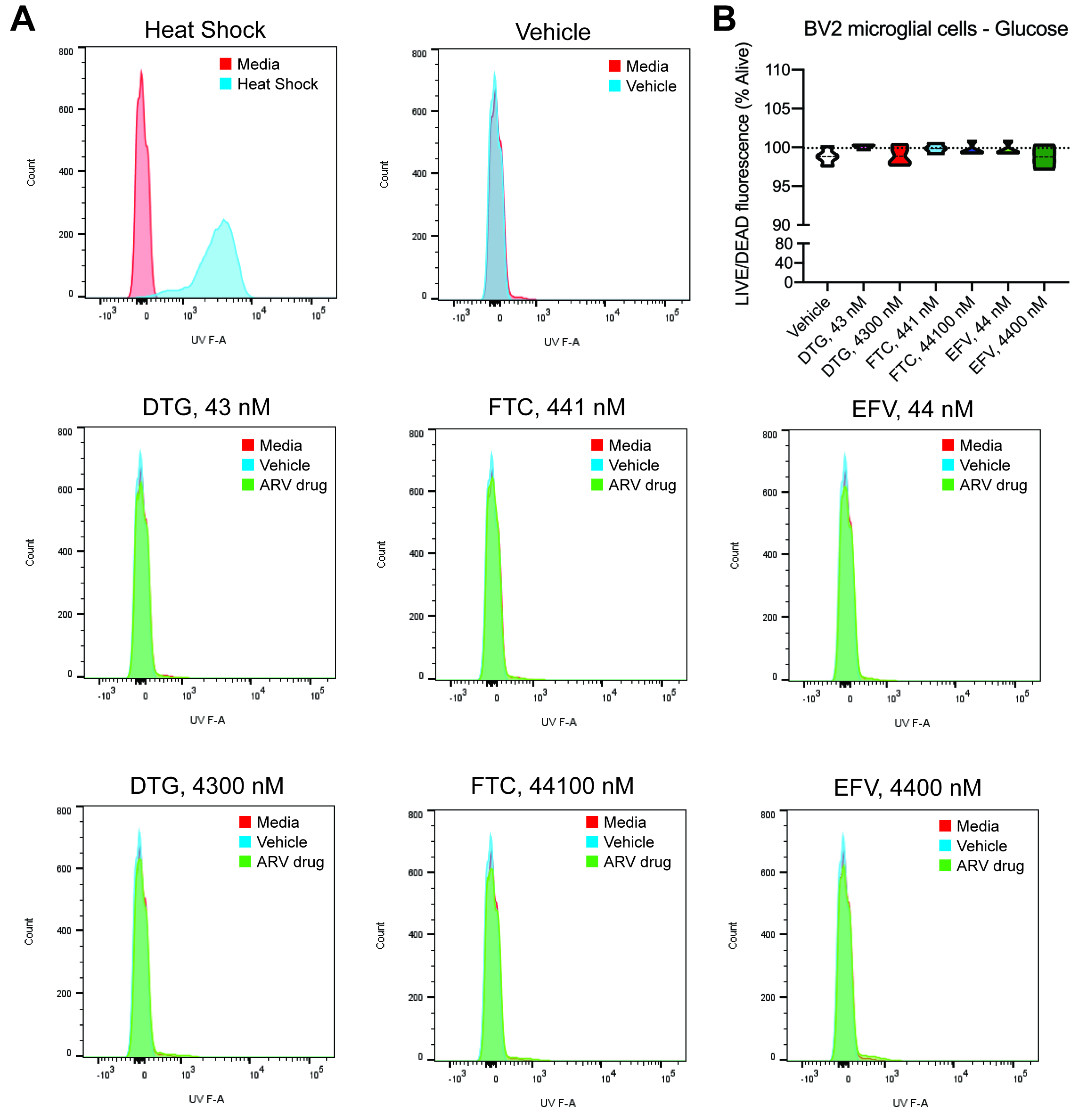

Supplement: Supplementary Figure 9 — BV2 cell viability unaltered by ARV treatment in the presence of glucose. BV2 cells were incubated for 24 hours with DTG, FTC, or EFV at the stated concentrations in glucose-containing media. Cell viability was determined using the LIVE/DEAD assay for flow cytometry and reported as the percent of viable (live) cells. n=4. [file Image_9.tif]

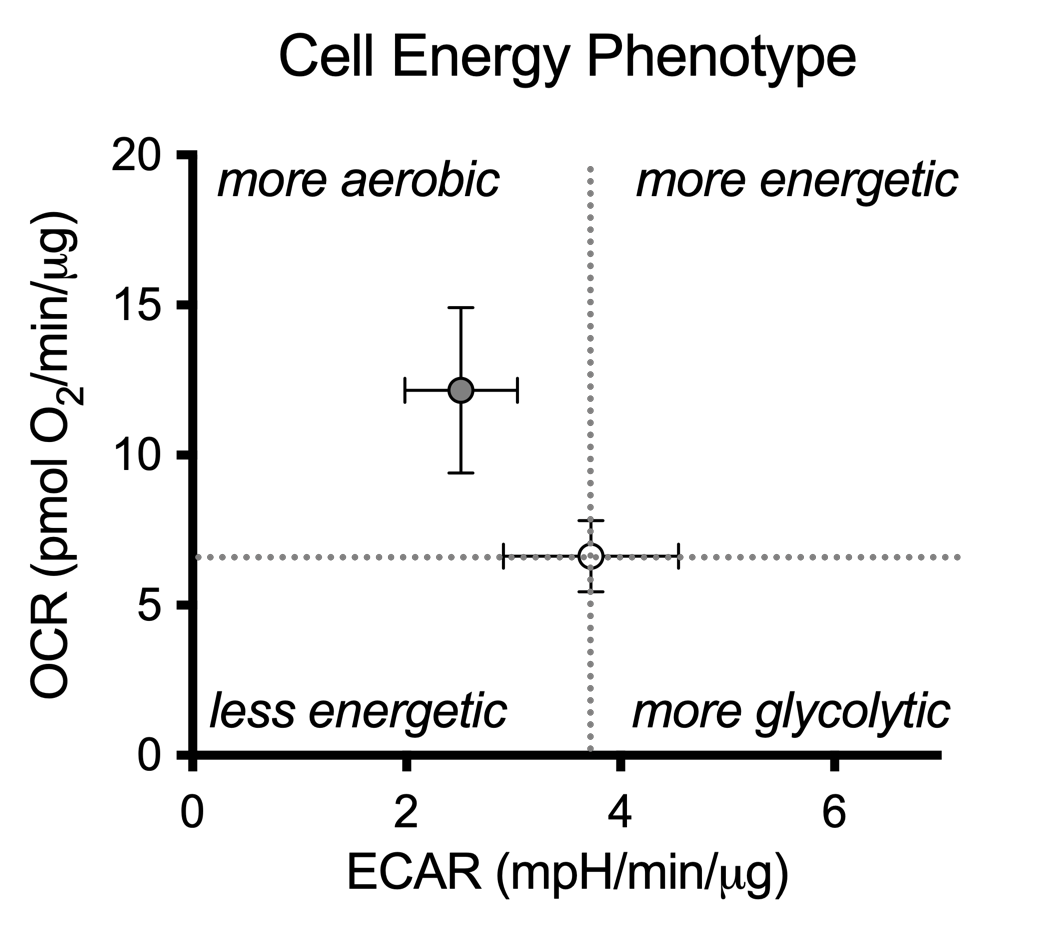

Supplement: Supplementary Figure 10 — Cell Energy Phenotype. Plot of baseline ECAR (glycolysis) and OCR (mitochondrial respiration) levels for HeLa (white dot) and BV2 (grey dot) cells. n=5. [file Image_10.tiff]
